# Imaging cerebral tryptophan metabolism in brain tumor-associated depression

**DOI:** 10.1186/s13550-015-0136-9

**Published:** 2015-10-17

**Authors:** Edit Bosnyák, David O. Kamson, Michael E. Behen, Geoffrey R. Barger, Sandeep Mittal, Csaba Juhász

**Affiliations:** Department of Pediatrics, Wayne State University, 3901 Beaubien Street, Detroit, MI 48201 USA; Department of Neurosurgery, Wayne State University, 4160 John R., Suite 930, Detroit, MI 48201 USA; Department of Oncology, Wayne State University, Detroit, MI USA; Department of Neurology, Wayne State University, 4201 St. Antoine, Detroit, MI 48201 USA; PET Center and Translational Imaging Laboratory, Children’s Hospital of Michigan, 3901 Beaubien Street, Detroit, MI 48201 USA; Karmanos Cancer Institute, Detroit, MI USA

**Keywords:** Positron emission tomography, Magnetic resonance imaging, Tryptophan, Serotonin, Kynurenine, Depression, Brain tumor, Cortical, Thalamus, Striatum

## Abstract

**Background:**

Depression in patients with brain tumors is associated with impaired quality of life and shorter survival. Altered metabolism of tryptophan to serotonin and kynurenine metabolites may play a role in tumor-associated depression. Our recent studies with alpha[^11^C]methyl-L-tryptophan (AMT)-PET in brain tumor patients indicated abnormal tryptophan metabolism not only in the tumor mass but also in normal-appearing contralateral brain. In the present study, we explored if tryptophan metabolism in such brain regions is associated with depression.

**Methods:**

Twenty-one patients (mean age: 57 years) with a brain tumor (10 meningiomas, 8 gliomas, and 3 brain metastases) underwent AMT-PET scanning. MRI and AMT-PET images were co-registered, and AMT kinetic parameters, including volume of distribution (VD’, an estimate of net tryptophan transport) and K (unidirectional uptake, related to tryptophan metabolism), were measured in the tumor mass and in unaffected cortical and subcortical regions contralateral to the tumor. Depression scores (based on the Beck Depression Inventory-II [BDI-II]) were correlated with tumor size, grade, type, and AMT-PET variables.

**Results:**

The mean BDI-II score was 12 ± 10 (range: 2–33); clinical levels of depression were identified in seven patients (33 %). High BDI-II scores were most strongly associated with high thalamic AMT K values both in the whole group (Spearman’s rho = 0.63, *p* = 0.004) and in the subgroup of 18 primary brain tumors (*r* = 0.68, *p* = 0.004). Frontal and striatal VD’ values were higher in the depressed subgroup than in non-depressed patients (*p* < 0.05); the group difference was even more robust when moderately/severely depressed patients were compared to patients with no/mild depression (frontal: *p* = 0.005; striatal: *p* < 0.001). Tumor size, grade, and tumor type were not related to depression scores.

**Conclusions:**

Abnormalities of tryptophan transport and metabolism in the thalamus, striatum, and frontal cortex, measured by PET, are associated with depression in patients with brain tumor. These changes may indicate an imbalance between the serotonin and kynurenine pathways and serve as a molecular imaging marker of brain tumor-associated depression.

**Trial registration:**

ClinicalTrials.gov NCT02367469

**Electronic supplementary material:**

The online version of this article (doi:10.1186/s13550-015-0136-9) contains supplementary material, which is available to authorized users.

## Background

Brain tumors comprise of about 90 % of all primary central nervous system tumors. In adults, meningiomas are the most common benign primary brain tumors, accounting for approximately 30 % of all brain tumors [1], while gliomas and metastatic brain tumors are the most frequently diagnosed malignancies in the brain parenchyma. Glioblastomas, the most malignant histologic type, comprise 65 % of all gliomas [2]. While brain tumors commonly manifest with seizures and focal neurological symptoms, cognitive impairment, mood disturbance, and depression are also common co-morbidities [3, 4]. In the general population, the prevalence estimates of major depressive disorder (MDD) were 14.9 % over a lifetime and 8.6 % over 12 months in the National Comorbidity Survey [5].

Most epidemiologic data related to brain-tumor-associated depression have encompassed primarily glioma patients, where the estimated prevalence of depression ranges from 6 to 93 % [3]. In a large study (*n* = 155), 20 % of newly diagnosed glioma patients fulfilled the diagnostic criteria of MDD. According to a systematic review, depression occurred independently of most tumor- or treatment-related variables, including brain tumor type, histologic grade, location, or various treatment modalities [6]. While depression in brain tumor patients is an important component of the quality of life, and, possibly, survival [6–8], many patients are neither properly diagnosed nor adequately treated for depression. Effective treatments for MDD include antidepressants (such as serotonin reuptake inhibitors) and psychotherapy. In a retrospective study of 160 glioblastoma patients, serotonin reuptake inhibitor treatment appeared to have some beneficial effect on survival after controlling for age and other prognostic factors [9]. In another study, presence of preoperative depression predicted worse survival in patients with low-grade gliomas [7]. In addition, preclinical studies suggested a direct chemotherapeutic effect of antidepressants in gliomas: both selective serotonin reuptake inhibitors (SSRIs) and tricyclic antidepressants induced glioma cell death by apoptosis in cell cultures [10, 11].

The precise pathophysiology of cancer-associated depression remains to be further elucidated. One theory involves an abnormal psychological reaction to overwhelming stress and loss [8, 12]. Another long-standing biological theory is related to abnormal serotonin levels due to a systemic shift between serotonin to kynurenine metabolism of tryptophan commonly referred to as the “kynurenine shunt” [13]. This effect has been described mostly in the context of major depression, where immune-mediated activation of indoleamine 2,3-dioxygenase (IDO), the initial key enzyme of the kynurenine pathway, and related decrease in serotonin synthesis have been considered key elements of the pathophysiology [14–16]. Increased pro-inflammatory cytokine levels have been linked to cancer-associated depression, partly via IDO activation, which may also lead to changes in brain serotonin levels [17–19]. Whether this mechanism plays a role in brain tumor-associated depression depression requires further study.

Molecular imaging of brain tryptophan metabolism and serotonergic function is a relatively new approach to study cerebral substrates of mood disorders and depression. Alpha[^11^C]methyl-L-tryptophan (AMT) is a positron emission tomography (PET) radiotracer accumulated in serotonergic neurons and is also a substrate of IDO of the kynurenine pathway [20–22]. AMT-PET studies in patients with MDD suggested focal abnormalities of brain serotonin synthesis [23–26]. Application of AMT-PET has also been studied in brain tumor imaging: AMT is accumulated in various brain tumors, and kinetic AMT-PET parameters are able to differentiate various brain tumor types, assess glioma proliferative activity, and estimate IDO and tryptophan 2,3-dioxygenase expression in both gliomas and meningiomas [27–32]. Interestingly, our recent studies in patients with brain tumors who underwent AMT-PET scan also demonstrated abnormal AMT uptake and kinetics in non-tumoral brain regions contralateral to the tumor [33, 34]. In patients with post-treatment malignant gliomas, altered AMT uptake in the contralateral cortex and thalamus was observed, and high thalamic uptake was a predictor of shorter survival [34].

In the present study, we used AMT-PET to explore the potential role of abnormal tryptophan metabolism in brain tumor-associated depression depression. We hypothesized that higher depression scores assessed at the time of the PET scan would be associated with abnormalities of AMT kinetic variables in the non-tumoral hemisphere. We also assessed if such changes were related to tumor type, histologic grade, or previous treatment. The overall goal of these studies was to determine if abnormal brain tryptophan metabolism, measured by PET, could be an imaging biomarker for brain tumor-associated depression.

## Methods

### Patient population

The study population included 21 patients (mean age: 57 years) with a brain tumor. Histopathology showed 10 World Health Organization (WHO) grade 1 meningiomas, 8 WHO grade 1–4 gliomas, and 3 brain metastases (1 breast cancer, 1 melanoma, and 1 non-small cell lung cancer) (Table 1). The four post-treatment patients had previous resective surgery with or without subsequent chemoradiation. Serial magnetic resonance imaging (MRI) follow-up demonstrated tumor progression or recurrence in all four patients; one patient also showed clinical progression. None of the 21 patients had a history of clinical depression, and they were not on any antidepressants at the time of the PET scan. All patients had a Karnofsky Performance Status score of 70 or higher. All patients were screened for depression on the day of the AMT-PET scan (see details below). Six of the 21 patients were on dexamethasone (which did not affect AMT kinetic values in non-tumoral brain in our previous study [33]), 10 patients had a history of seizure(s), and 12 were on antiepileptic medication at the time of the AMT-PET scan. The study was approved by the Institutional Review Board of Wayne State University (#091602MP2F(5R)), and written informed consent was obtained from all participants.

**Table 1:** Clinical data, BDI-II depression scores, imaging and histopathology data of the patients, who are listed in the order of decreasing BDI-II scores

	Gender	Age (years)	BDI-II score	New/rec	Tumor type	Grade	Area max (cm^2^)	Location	Side
1	F	55	33	New	Glioma	4	12.9	FP	R
2	F	22	30	New	Meningioma	2	8.5	F	R
3	M	52	28	New	Meningioma	1	12.2	Brainstem	M
4	M	67	22	New	Metastasis		1.8	F	R
5	M	70	20	New	Metastasis		17.7	TP	L
6	M	63	17	Rec	Glioma	2	43.6	FT	R
7	M	48	14	New	Glioma	3	25.3	F	L
8	F	49	13	New	Meningioma	1	9.6	F	L
9	M	59	11	New	Meningioma	1	4.6	Medulla	M
10	F	37	9	New	Glioma	2	11.8	F	L
11	M	58	8	Rec	Meningioma	2	8.7	P	R
12	M	25	6	New	Glioma	1	1	O	R
13	M	46	6	Rec	Glioma	4	2.6	PO, T	R
14	F	60	6	New	Metastasis		1.1	F	L
15	F	84	5	New	Meningioma	1	13.8	F	L
16	F	70	5	New	Glioma	4	1.6	T	R
17	M	60	3	New	Meningioma	1	13.2	Olfactory	M
18	F	62	3	New	Meningioma		6.2	FT	L
19	M	68	3	New	Meningioma		5.1	R lat clivus	R
20	M	79	2	New	Glioma	4	6.1	T	L
21	M	68	2	New	Meningioma	1	9.1	F	L

*BDI-II* Beck Depression Inventory-II, *M* male, *F* female, *new/rec* newly diagnosed/recurrent tumor, *F* frontal, *T* temporal, *P* parietal, *O* occipital, *R* right, *L* left, *M* midline, *lat* lateral

### AMT-PET scanning protocol

Fifteen of the 21 AMT-PET studies were performed using a GE Discovery STE PET/CT scanner (GE Medical Systems, Milwaukee, WI) and six scans were performed using a Siemens EXACT/HR whole-body positron emission tomograph, both located at the PET Center of the Children’s Hospital of Michigan in Detroit. Both scanners have a 15-cm field of view and generate 47 image planes with a slice thickness of 3 mm. The reconstructed image in-plane resolution obtained is 7.5 ± 0.4 mm at full-width half-maximum (FWHM) and 7.0 ± 0.5 mm in the axial direction (reconstruction parameters: Hanning filter with 1.26 cycles/cm cutoff frequency) for the Siemens scanner. The GE scanner has a similar resolution, with FWHM of 7.5 mm (isotropic), and images from this scanner were reconstructed with an iterative reconstruction (2 iterations, 16 subsets, 8-mm axial smoothing). The AMT tracer was synthesized using a high-yield procedure as previously outlined [35]. The procedure for AMT-PET scanning has been described in detail elsewhere [27]. In short, following 6 h of fasting, a slow bolus of AMT (3.7 MBq/kg) was injected intravenously over 2 min. For collection of timed blood samples, a second intravenous line was established. In the initial 20 min of the scan following tracer injection, a dynamic PET scan of the heart was performed to obtain the blood input function from the left cardiac ventricle non-invasively. The blood input function was continued beyond these initial 20 min by using venous blood samples (0.5 mL/sample, collected at 20, 30, 40, 50, and 60 min after AMT injection). At 25 min after tracer injection, a dynamic emission scan of the brain (7 × 5 min) was obtained. Measured attenuation correction, scatter, and decay correction were applied to all PET images.

### MRI protocol

Diagnostic MRI scans with routine post-gadolinium T1 (T1-Gad), T2-weighted, and fluid-attenuated inversion recovery (FLAIR) axial images acquired closest in time (typically within 2 weeks) to the AMT-PET were used in the study. MRI was performed on one of three 3T scanners using similar parameters: (i) Siemens MAGNETOM Trio TIM, (ii) GE Signa HDxt, or (iii) Philips Achieva TX.

### AMT-PET image processing and analysis

**Fig. 1:**
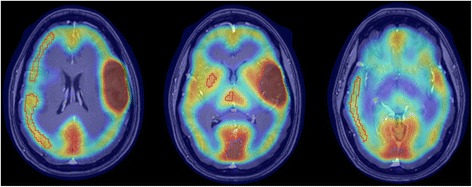
AMT-PET/T1-Gad MRI fusion images of a patient (#10) with left frontal WHO grade 2 glioma. Contralateral frontal, parietal, and temporal cortical, as well as thalamic and striatal regions of interest used for analysis, are outlined in *red*

For visualization of AMT uptake in the brain, averaged activity images 30–55 min post-injection were created and converted to an AMT standardized uptake value (SUV) image. For quantification of AMT accumulation, a Patlak graphical analysis was performed using the dynamic brain PET images and blood input function [27, 36]. This approach provides two main kinetic parameters. The y-intercept of the Patlak plot yields the tracer’s apparent volume of distribution (VD’), which is tightly correlated with VD values derived from compartmental analysis [21, 27, 37]; AMT VD’ values are indicative of the net transport of tryptophan into the tissue of interest (tumor or cortex). The slope of the Patlak plot (K-complex or K) reflects the unidirectional uptake of the tracer into the tissue and correlates with tryptophan metabolism via the serotonin synthesis pathway in cortex [21, 22]. In brain tumors, with no evidence of serotonin synthesis, the most likely mechanism of an increase in AMT K-complex is tumoral accumulation in the form of either unmetabolized AMT or kynurenine metabolites. The advantages and limitations of using these kinetic AMT-PET parameters have been discussed previously [21, 27, 30]. For image analysis, the 3D Slicer 3.6.3 software suite was used (Brigham and Women’s Hospital, Inc.) as described previously [32, 34, 38, 39]. First, a transformation matrix was created by co-registration of the summed AMT-PET images to the T1-Gad volumetric image volumes (magnetization-prepared rapid gradient-echo [MPRAGE] protocol on Siemens or MPRAGE-equivalent sequence on GE and Philips scanners) as well as FLAIR images using the Fast Rigid Registration module. This transformation matrix was then applied to the summed AMT-PET image and to the dynamic AMT-PET images loaded via the 4D Image module of the 3D Slicer. Following fusion of the summed AMT-PET with MR images, the largest orthogonal diameters as well as maximum tumor area on T1-Gad (or T2/FLAIR images, if no enhancement was seen) were measured. Regions of interest (ROIs) were drawn on the tumor mass in tumor regions with contrast enhancement and/or T2/FLAIR signal changes on MRI. In addition, cortical and subcortical regions, contralateral to the tumor, were delineated and analyzed as described recently [34]; for the three tumors close to the midline, the tumor side was defined as the side with the larger tumor mass. In brief, regions of interest were segmented manually using the axial view of the PET/MRI fusion images (Fig. 1). The ROIs included gray matter voxels with AMT uptake: a single plane for the thalamus and striatum with the largest axial diameter and multiple planes (one to two planes apart) for the cortical ROIs including the frontal (three planes), temporal (two planes), and parietal (two planes) lobes. None of these ROIs showed abnormal contrast-enhancement or FLAIR signal on co-registered MR images. Similar to our previous study, the occipital cortex, which often shows high physiologic AMT uptake, was not included in the analyses. All tumoral and contralateral cortical and subcortical ROIs were applied on the co-registered AMT SUV as well as dynamic PET images, and AMT SUV as well as K and VD’ values from the Patlak analysis was obtained for each region.

### Assessment of depression

Symptoms of depression were assessed using the Beck Depression Inventory, 2nd Edition (BDI-II) [40]. The BDI-II is a self-reported measure that identifies the presence and severity of symptoms of depression. There are 21 items on the BDI-II each rated on a four-point Likert scale (0–3). The measure yields a total score, and the cutoffs for depression severity are as follows: 0–13 = no/minimal depression, 14–19 = mild depression, 20–28 = moderate depression, and 29–63 = severe depression. The psychometric properties of the scale have been well established, and the measure is widely used with both clinical and research samples including different cancer patient groups [6–8, 41, 42].

### Statistical analysis

First, BDI-II depression scores were compared among three tumor types (gliomas, meningiomas, metastases) using the Kruskal-Wallis test. BDI-II depression scores were also correlated with tumor type, histologic grade, tumor size, and AMT-PET variables in the whole group as well as subgroups (such as patients with a primary brain tumor, patients with an intraaxial tumor [glioma or metastasis], and patients with a newly diagnosed tumor) using Spearman’s rank correlations. AMT kinetic variables were compared between depressed (BDI-II score >13) and non-depressed patients, as well as between moderately/severely depressed patients (BDI-II score ≥20) vs. the rest of the patients, using the Mann-Whitney *U* test. Statistical analyses were performed using IBM SPSS Statistics, version 21. A *p* value less than 0.05 was considered to be significant.

## Results

### Whole group analysis

Mean BDI-II total score was 12 ± 10 (range: 2–33). A total of seven patients (33 %) had scores indicating various levels of depression: five patients (24 %) had moderate or severe depression (BDI-II score ≥20), and two additional patients had mild depression (Table 1). Mean BDI-II scores (gliomas: 12 ± 10, meningiomas: 11 ± 10, metastases: 16 ± 9) were not different across tumor types, *p* = 0.47.

**Fig. 2:**
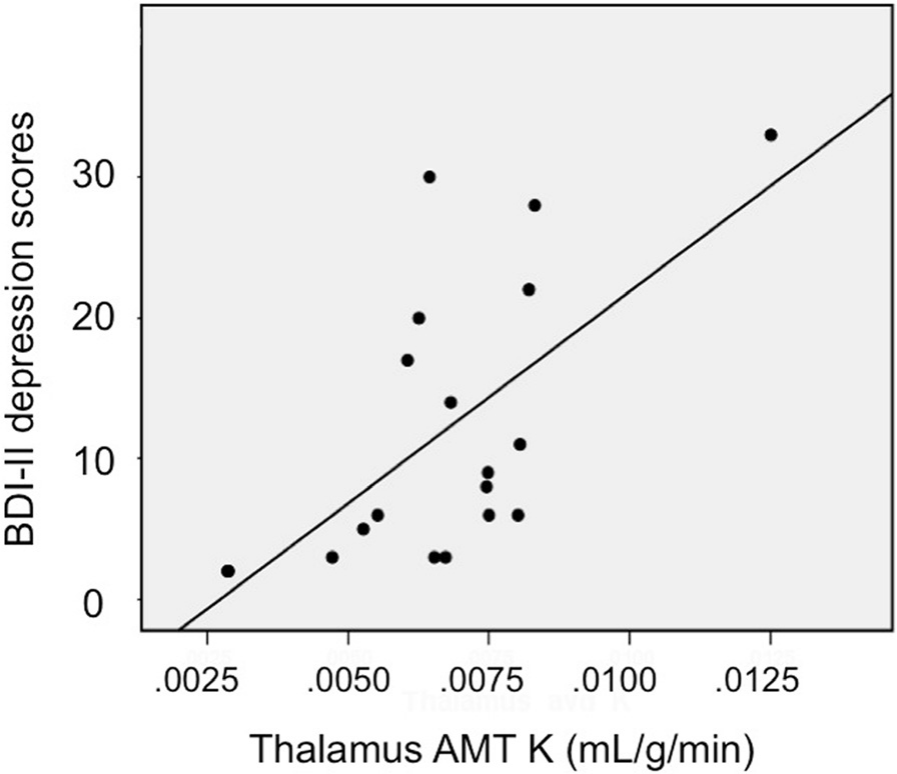
Positive correlation between thalamus AMT K values and BDI-II depression scores (Spearman’s rho = 0.63, *p* = 0.004)

**Fig. 3:**
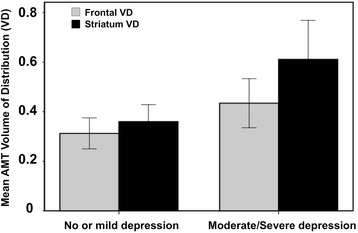
Comparison of AMT-PET variables in patients with no/mild depression vs. moderate/severe depression. Frontal and striatal AMT volume of distribution (VD’) values were significantly higher in patients with moderate/severe depression (frontal VD’: 0.43 vs. 0.31, *p* = 0.005; striatal VD’: 0.61 vs. 0.35, *p* < 0.001)

In the whole group, the BDI-II depression scores showed a significant positive correlation with thalamic and temporal cortical K values, with the most robust correlations found for the thalamic values (*r* = 0.63; *p* = 0.004), that remained significant even after Bonferroni correction for multiple correlations (Additional file 1: Table S1, Fig. 2). In comparison of depressed (*n* = 7) vs. not depressed patients, frontal and striatal VD’ values were significantly higher in the depressed subgroup (0.40 ± 0.10 vs. 0.31 ± 0.06, *p* = 0.017 and 0.53 ± 0.19 vs. 0.36 ± 0.07, *p* = 0.035, respectively). The difference was even more robust when moderately/severely depressed patients (*n* = 5) were compared to patients with no/mild depression (frontal VD’: 0.43 ± 0.10 vs. 0.31 ± 0.06, *p* = 0.005; striatal VD’: 0.61 ± 0.16 vs. 0.35 ± 0.07, *p* < 0.001) (Fig. 3). Tumor size, tumor grade (in the primary brain tumors), tumor type, tumoral AMT-PET variables, and AMT SUVs in any structures were not related to the depression scores.

### Subgroup analysis

In the 18 patients with primary tumors (gliomas and meningiomas), thalamic as well as temporal and parietal cortical K values showed significant positive correlations with BDI-II depression scores (Additional file 1: Table S2); the most robust correlation was again found with thalamic K values (*r* = 0.68; *p* = 0.004), similar to the whole-group results. In the 12 patients with an intraaxial tumor, only thalamic K values showed a positive correlation with the BDI-II depression scores (*r* = 0.65, *p* = 0.03). Finally, in the pretreatment subgroup (*n* = 18), thalamic AMT K values showed a positive correlation with the BDI-II depression scores (*r* = 0.61; *p* = 0.013).

## Discussion

Although there have been past studies showing focal brain abnormalities of tryptophan metabolism in patients with depression not associated with cancer [24–26], this is the first study to assess imaging correlates of cerebral tryptophan metabolism in brain tumor-associated depression depression. Similar to previous studies, we noted a high rate of depression (one third in our cohort) in a group of patients with a brain tumor, who were not previously diagnosed and treated for depression clinically. Our main finding is that higher depression scores were related to variations in tryptophan kinetic variables in several cortical and subcortical structures contralateral to the tumors in brain regions showing no apparent MRI abnormalities. Specifically, depressed patients showed higher AMT VD values (suggesting higher tryptophan transport) in the frontal cortex and striatum, two key components of the fronto-striatal network, previously implicated in depressive symptoms [43]. These increases are likely related to the upregulation of the kynurenine pathway, thus leading to an imbalance between tryptophan metabolism via the serotonin and kynurenine pathways. Such an imbalance may play a role in brain tumor-associated depression depression.

In previous AMT-PET studies in patient groups with depression not associated with cancer, decreased tryptophan metabolism was found in the anterior cingulate cortex as well as the mesial temporal cortex in MDD patients [23] and in the frontal cortex (orbital and ventromedial prefrontal regions) in suicide attempters [24]. Another study reported increased tryptophan metabolism in the prefrontal cortex after combined antidepressant treatment (SSRI plus 5-HT_1A_ receptor antagonist pindolol) in MDD patients [26]. Frey et al. [25] also reported regional gender differences in MDD, with higher focal tryptophan metabolism in multiple brain regions, such as the anterior cingulate cortex, inferior frontal gyrus, parahippocampal gyrus, precuneus, superior parietal lobule, and occipital lingual gyrus, in women. However, previous imaging studies of cerebral structural or functional correlates of cancer-associated depression are limited. 2-deoxy-2[^18^F]fluoro-D-glucose (FDG)-PET studies showed decreased glucose metabolism in limbic structures, such as the anterior and posterior cingulate gyri, basolateral frontal cortices, dorsolateral prefrontal cortex (DLPFC), temporo-parietal cortices, insular cortex, and also in the basal ganglia in depressed patients with different types of cancer (malignant melanoma, malignant lymphoma, cervical cancer, pancreatic cancer, and colon cancer) [44, 45]. A subsequent FDG-PET study, in a heterogeneous extracranial cancer population with predisposition to onset of depression, showed decreased glucose metabolism in the right medial frontal gyrus but increased metabolism in the right anterior and posterior cingulate gyri, left subcallosal gyrus, and left caudate nucleus [46]. Increased metabolism in the subgenual anterior cingulate cortex was also reported in pancreatic cancer patients with minor and major depression [47]. One study investigating FDG uptake changes during an attention task in breast cancer patients found decreased glucose metabolism in the bilateral DLPFC and right premotor cortex, as well as in the left inferior parietal lobe in depressed patients [48]. Altogether, these glucose PET findings showed metabolic abnormalities in some of the brain structures found to show depression-related variations in our present study, including fronto-temporal cortices and the basal ganglia. Also consistent with this, FDG-PET studies performed in patients with MDD (not associated with cancer) showed decreased brain metabolism in similar brain regions, including the prefrontal cortex, basal ganglia, and temporo-parietal cortices [49, 50]. In addition, increased glucose metabolism in the orbital cortex, amygdala, and thalamus was reported in some studies [51, 52]. Overall, these data strongly suggest that the main neural substrates of depression are similar in cancer patients and those with major depression.

The abovementioned structures, showing metabolic changes in depressed patients, are parts of specific brain circuits that play a role in depressive symptoms, such as the fronto-striatal and limbic circuits. A functional MRI (fMRI) study reported a possible role of fronto-striatal circuits in MDD [43] by showing attenuated functional connectivity between the ventral striatum and both ventromedial prefrontal cortex and subgenual anterior cingulate cortex and also stronger connectivity between dorsal caudate and dorsal prefrontal cortex, which correlated with the severity of depression. Another fMRI study showed increased network functional connectivity in the subgenual cingulate, thalamus, and orbitofrontal cortex in depressed patients [53]. Interestingly, a histopathology study from postmortem issues demonstrated elevated total neuron number in the limbic thalamus region in MDD patients [54]. Also, a postmortem study in suicide patients with MDD demonstrated increased microglia density in the DLPFC, anterior cingulate cortex, and mediodorsal thalamus [55]. This latter finding suggested active immunological processes and neuroinflammation in some of the brain regions whose tryptophan kinetic parameters showed a correlation with depression scores in our study.

One concern with the use of the BDI-II in subjects with a somatic disease is that the total score may, at least partially, reflect the disease severity rather than clinical depression. For example, a previous study demonstrated that the somatic subscale score may be better attributed to the severity of the cancer disease, rather than the severity of depression [56]. In a recent study of brain tumor patients, the somatic subscale score provided the strongest contribution to the total variance of the Beck score [57]. Another study showed that elderly patients with co-morbidities have higher scores on the somatic subscale than younger subjects [58]. In our study, an exploratory analysis showed that the somatic subscale scores had the strongest correlation with thalamic AMT uptake, while the cognitive-affective scores also correlated with fronto-striatal tryptophan kinetic variables (data not shown). Although this observation will need to be addressed in a larger study, it is possible that tryptophan metabolic changes in the thalamus are more related to the cancer-associated somatic symptoms, while the fronto-striatal tryptophan changes are more tightly associated with clinical depression. This latter is further supported by the finding that depressed brain tumor patients showed higher AMT VD values in the frontal cortex and striatum without significant differences in the thalamus.

The exact cause of higher tryptophan kinetic parameters measured in non-tumoral brain regions of depressed brain tumor patients remains to be determined. A plausible mechanism would be higher tryptophan demand due to increased tryptophan metabolism via the kynurenine pathway. IDO, the rate-limiting enzyme of this pathway, is upregulated by pro-inflammatory cytokines, which are known to increase as a result of chronic inflammatory responses in various inflammatory disorders and cancer; a main source of cytokine release in the brain is derived from activated microglia, and this mechanism is implicated in the pathophysiology of cancer-associated depression [19, 59–61]. In addition, tumor treatment effects, especially radiation, may also induce brain inflammatory responses [62]. This is unlikely to be the main mechanism in our study, because the majority of the patients had no previous treatment, and the key findings remained significant in the pretreatment subgroup. A third possibility for increased tryptophan kinetics in remote brain regions could be the presence of microscopic tumor infiltration, not visualized on MRI. This could occur in some infiltrative gliomas or brain metastases, but not in patients with meningiomas; therefore, this is also an unlikely explanation. The observed variations in tryptophan kinetics may be also modulated by simultaneous changes in serotonin synthesis (often seen as decreased in depression). This makes the interpretation of our PET findings more complicated, since AMT can track both the kynurenine and serotonin pathways [22]. Additional imaging studies, employing radioligands specific for the kynurenine or serotonin pathways, could clarify this issue. Such ligands, such as radiotracers tracking IDO activity, are being currently developed [63, 64]. The role of neuroinflammation in brain tumor-associated depression depression could also be further studied by molecular imaging approaches targeting activated microglia, e.g., by using PET radioligands for the translocator protein [65].

This study has some limitations. The study population was small; therefore, potential tumor-type-specific effects could not be completely excluded (although major effects were not detected). Also, our analysis was confined to a limited number of brain regions and did not include some potentially relevant structures, such as the amygdala. However, spatial resolution of AMT-PET is limited, which makes reliable assessment of detailed tracer kinetics in small regions difficult. Another potential confounding factor could be the effect of brain volume loss on PET metabolic values. Volumetric MRI studies indeed demonstrated variable volume decreases in a number of cortical and subcortical brain regions in depressed patient populations [66–70], although the results were inconsistent. In a recent study of MDD patients, reduced striatal volume was inversely associated with serum kynurenine and kynurenine/tryptophan ratios [71]. Although brain volumetric data on cancer-associated depression are scarce, the higher tryptophan kinetic parameters in depressed patients in our study cannot be explained by volume loss, which would likely result in lower (rather than increased) tracer uptake in these structures. Finally, we did not have AMT kinetic data in an age-matched healthy control group to assess if the observed, depression-related variations of AMT-PET parameters in the studied regions were outside of the physiologic range. Thus, these study results will need to be further confirmed in a larger patient population and compared to data of healthy control subjects.

## Conclusions

These results demonstrate that tryptophan transport and metabolism in the thalamus, striatum, and fronto-temporal cortex are associated with depression in patients with a brain tumor. The observed imaging abnormalities may indicate an imbalance between the serotonin and kynurenine pathways. Altered tryptophan metabolism in non-tumoral brain, measured by PET, may be a novel imaging marker of brain tumor-associated depression.

## Additional file

Additional file 1:
**BDI-II scores and AMT kinetic parameters.** **Table S1.** Correlations (Spearman’s rank) between AMT kinetic parameters and BDI-II scores. **Table S2.** Correlations (Spearman’s rank) between BDI-II scores and AMT kinetic parameters in patients with primary brain tumors

## Abbreviations

AMT: alpha[^11^C]methyl-L-tryptophan
BDI-II: Beck Depression Inventory-II
DLPFC: dorsolateral prefrontal cortex
FDG: 2-deoxy-2[^18^F]fluoro-D-glucose
FLAIR: fluid-attenuated inversion recovery
FWHM: full-width half-maximum
IDO: indoleamine 2,3-dioxygenase
MDD: major depressive disorder
MPRAGE: magnetization-prepared rapid gradient-echo
MRI: magnetic resonance imaging
PET: positron emission tomography
ROI: region of interest
SUV: standardized uptake value
VD’: volume of distribution’
WHO: World Health Organization
