# Supplementary material for: Imaging cerebral tryptophan metabolism in brain tumor-associated depression
Source: EJNMMI Res. 2015 Oct 17;5:56. doi: 10.1186/s13550-015-0136-9 (PMC4608955; doi:10.1186/s13550-015-0136-9)
Supplement: Additional file 1: — BDI-II scores and AMT kinetic parameters. Table S1. Correlations (Spearman’s rank) between AMT kinetic parameters and BDI-II scores. Table S2. Correlations (Spearman’s rank) between BDI-II scores and AMT kinetic parameters in patients with primary brain tumors [file 13550_2015_136_MOESM1_ESM.docx › ESM/13550_2015_136_MOESM1_ESM.docx]

**Supplementary file**

**Table S1**. Correlations (Spearman’s rank) between AMT kinetic parameters and BDI-II scores. VD’: volume of distribution; K: unidirectional uptake rate on AMT-PET

| **Brain region** | | **Spearman’s rho** | ***p* value** |
| --- | --- | --- | --- |
| **Thalamus** | |  |  |
| *VD’* | | 0.12 | 0.62 |
| *K* | | 0.63 | 0.004* |
| **Striatum** | |  |  |
| *VD’* | | 0.37 | 0.11 |
| *K* | | 0.17 | 0.49 |
| **Frontal cortex** | |  |  |
| *VD’* | | 0.42 | 0.08 |
| *K* | | 0.19 | 0.42 |
| **Temporal cortex** | |  |  |
| *VD’* | | 0.27 | 0.27 |
| *K* | | 0.46 | 0.048 |
| **Parietal cortex** | |  |  |
| *VD’* | | 0.28 | 0.25 |
| *K* | 0.36 | 0.12 |  |

*: Value is significant after Bonferroni correction for multiple correlations

**Table S2**. Correlations (Spearman’s rank) between BDI-II scores and AMT kinetic parameters in patients with primary brain tumors

| **Brain region** | **Spearman’s rho** | ***p* value** |
| --- | --- | --- |
| **Thalamus** |  |  |
| *VD’* | 0.13 | 0.62 |
| *K* | 0.68 | 0.004* |
| **Striatum** |  |  |
| *VD’* | 0.15 | 0.58 |
| *K* | 0.39 | 0.13 |
| **Frontal cortex** |  |  |
| *VD’* | 0.29 | 0.28 |
| *K* | 0.39 | 0.13 |
| **Temporal cortex** |  |  |
| *VD’* | 0.14 | 0.60 |
| *K* | 0.64 | 0.008 |
| **Parietal cortex** |  |  |
| *VD’* | 0.12 | 0.65 |
| *K* | 0.57 | 0.02 |

*: Value is significant after Bonferroni correction for multiple correlations
